# Relationship between brain function (aEEG) and brain structure (MRI) and their predictive value for neurodevelopmental outcome of preterm infants

**DOI:** 10.1007/s00431-018-3166-2

**Published:** 2018-05-22

**Authors:** Britta Hüning, Tobias Storbeck, Nora Bruns, Frauke Dransfeld, Julia Hobrecht, Julia Karpienski, Selma Sirin, Bernd Schweiger, Christel Weiss, Ursula Felderhoff-Müser, Hanna Müller

**Affiliations:** 1 Department of Pediatrics I, Neonatology, Pediatric Intensive Care, Pediatric Neurology, University Hospital Essen, University Duisburg-Essen, Hufelandstr. 55, 45147 Essen, Germany; 2 Institute of Diagnostic and Interventional Radiology and Neuroradiology, University Hospital Essen, University Duisburg-Essen, Hufelandstr. 55, 45147 Essen, Germany; 3 Department of Medical Statistics and Biomathematics, University Hospital Mannheim, University of Heidelberg, Ludolf-Krehl-Straße 13-17, 68167 Mannheim, Germany; 4 Division of Neonatology and Pediatric Intensive Care, Department of Pediatrics and Adolescent Medicine, Friedrich-Alexander-University of Erlangen-Nürnberg, Loschgestr. 15, 91054 Erlangen, Germany

**Keywords:** aEEG, MRI, Immature preterm infants, Neurodevelopmental outcome

## Abstract

To improve the prediction of neurodevelopmental outcome in very preterm infants, this study used the combination of amplitude-integrated electroencephalography (aEEG) within the first 72 h of life and cranial magnetic resonance imaging (MRI) at term equivalent age. A single-center cohort of 38 infants born before 32 weeks of gestation was subjected to both investigations. Structural measurements were performed on MRI. Multiple regression analysis was used to identify independent factors including functional and structural brain measurements associated with outcome at a corrected age of 24 months. aEEG parameters significantly correlated with MRI measurements. Reduced deep gray matter volume was associated with low Burdjalov Score on day 3 (*p* < 0.0001) and day 1–3 (*p* = 0.0012). The biparietal width and the transcerebellar diameter were related to Burdjalov Score on day 1 (*p* = 0.0111; *p* = 0.0002). The final multiple regression analysis revealed independent predictors of neurodevelopmental outcome: intraventricular hemorrhage (*p* = 0.0060) and interhemispheric distance (*p* = 0.0052) for mental developmental index; Burdjalov Score day 1 (*p* = 0.0201) and interhemispheric distance (*p* = 0.0142) for psychomotor developmental index.

*Conclusion*: Functional aEEG parameters were associated with altered brain maturation on MRI. The combination of aEEG and MRI contributes to the prediction of outcome at 24 months.

**Table:** 

**What is Known:** • *Prematurity remains a risk factor for impaired neurodevelopment*. • *aEEG is used to measure brain activity in preterm infants and cranial MRI is performed to identify structural gray and white matter abnormalities with impact on neurodevelopmental outcome*.	
**What is New:** • *aEEG parameters observed within the first 72 h of life were associated with altered deep gray matter volumes, biparietal width, and transcerebellar diameter at term equivalent age*. • *The combination of aEEG and MRI contributes to the prediction of neurodevelopmental outcome at 2 years of corrected age in very preterm infants*.

**Electronic supplementary material:**

The online version of this article (10.1007/s00431-018-3166-2) contains supplementary material, which is available to authorized users.

## Introduction

Progress in perinatal medicine led to increased survival rates of prematurely born children in recent decades. Nevertheless, the range of up to 50% of former very immature preterm infants with behavioral or neurological impairment persisting into adulthood reveals the potential to improve neurodevelopmental outcome [1, 23, 25, 30]. Next to the development of innovative neuroprotective strategies in postnatal treatment, the identification of children at high risk of later developmental impairment might first of all help the individual but also society to support normal everyday life. In addition, early predictors might serve as useful biomarkers for clinical interventions.

Both amplitude-integrated electroencephalography (aEEG) and magnetic resonance imaging (MRI) at term equivalent age (TEA) have been independently used for the identification of predictors for prognosis [18, 37]. aEEG proved itself in practice in neonatal intensive care units (NICUs) for continuous monitoring of cerebral function following birth asphyxia. There is growing evidence that early postnatal aEEG in preterm born neonates correlates with neurodevelopmental outcome [15, 21]. In addition, the combination of different classification systems of aEEG patterns [11, 14] to assess outcome seems reasonable [10].

The benefit of MRI at TEA is to reveal subtle injury patterns that are difficult to detect on cranial ultrasound such as white matter injury, cerebellar hemorrhages (CBH), and altered brain maturation [29, 35, 39]. Qualitative MRI analysis and advanced MRI techniques identified structural gray and white matter abnormalities and cerebellar injury with impact on neurodevelopmental outcome [27, 34, 38, 39]. However, most of these methods remain object of research and appear to be too complex for daily routine. Kidokoro et al. suggested a scoring system and simple brain metrics to characterize brain injury and impaired development in very preterm infants [20].

**Fig. 1:**
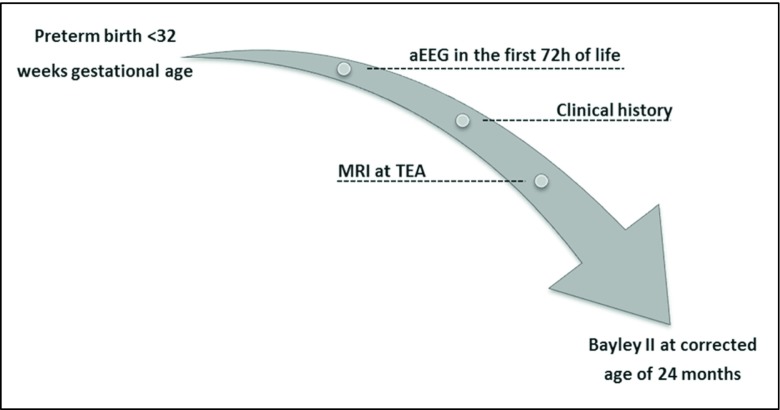
Schematic illustration of the study. TEA = term equivalent age

The aim of this single-center study was to investigate the relationship between brain function, maturation, and brain structure of very preterm infants by combining early postnatal aEEG monitoring and MRI at TEA in order to improve prediction of neurodevelopmental outcome at 24 months’ corrected age (Fig. 1).

## Methods

### Patients

All preterm infants enrolled in this retrospective study were born at the University Hospital Essen before 32 weeks of gestation from March 2011 to December 2012 and underwent both aEEG of at least 4 hours (h) of representative quality per day within the first 72 h of life and MRI at TEA. Exclusion criteria were severe abnormalities (congenital malformations, chromosomal abnormalities, and genetic disorders). Ethical approval of the local Ethics committee of the University of Essen was obtained (15-6522-BO; 12-4981-BO).

### aEEG recordings

aEEG monitoring was performed within the first 72 h of life using needle electrodes and a two-channel EEG on BRM2/BRM3 monitors (BrainZ Instruments, New Zealand). Electrodes were placed by nursing staff as early as possible after birth corresponding to positions C3, P3, C4, and P4 of the 10–20 system with a reference electrode on the back. aEEGs were interpreted in the single-channel mode by two investigators (N.B. and H.M.) using the Burdjalov scoring system [11]. The first 4 h within each day showing continuously good quality (no artifacts, impedance < 15 kΩ, no sedation or opioids within the last 12 h) were selected for independent analysis. Results were collected for each of the first 3 days of life (Burdjalov Score = BS 1,2,3) and added up to a total Burdjalov Score 1–3 (BS 72 h).

### MRI acquisition

MRI scans were performed after parental consent on a 3 Tesla MR scanner (Magnetom Skyra, Siemens Healthcare, Erlangen, Germany) using a MR-compatible incubator with dedicated 8-channel neonatal head coil (LMT Medical Systems nomag IC, Lübeck, Germany) as previously described [31]. A minority of infants (15 in total, 39%) were sedated using chloral hydrate (25- to 50-mg/kg bodyweight). Standard imaging protocols with an average length of 15 min included transversal T2-weighted turbo spin echo, T1-weighted 3D fast low-angle shot (FLASH), susceptibility-weighted (SWI), and diffusion-weighted (gradients: b0, b700, b1000) imaging. Qualitative analysis and scoring was done in consensus by two radiologists (S.S., B.S.) and a neonatologist (B.H.) blinded to the clinical course. Quantitative analysis including volumetry was performed manually by a pediatrician (T.S.) supervised by the radiologists mentioned above.

### MRI analysis

**Fig. 2:**
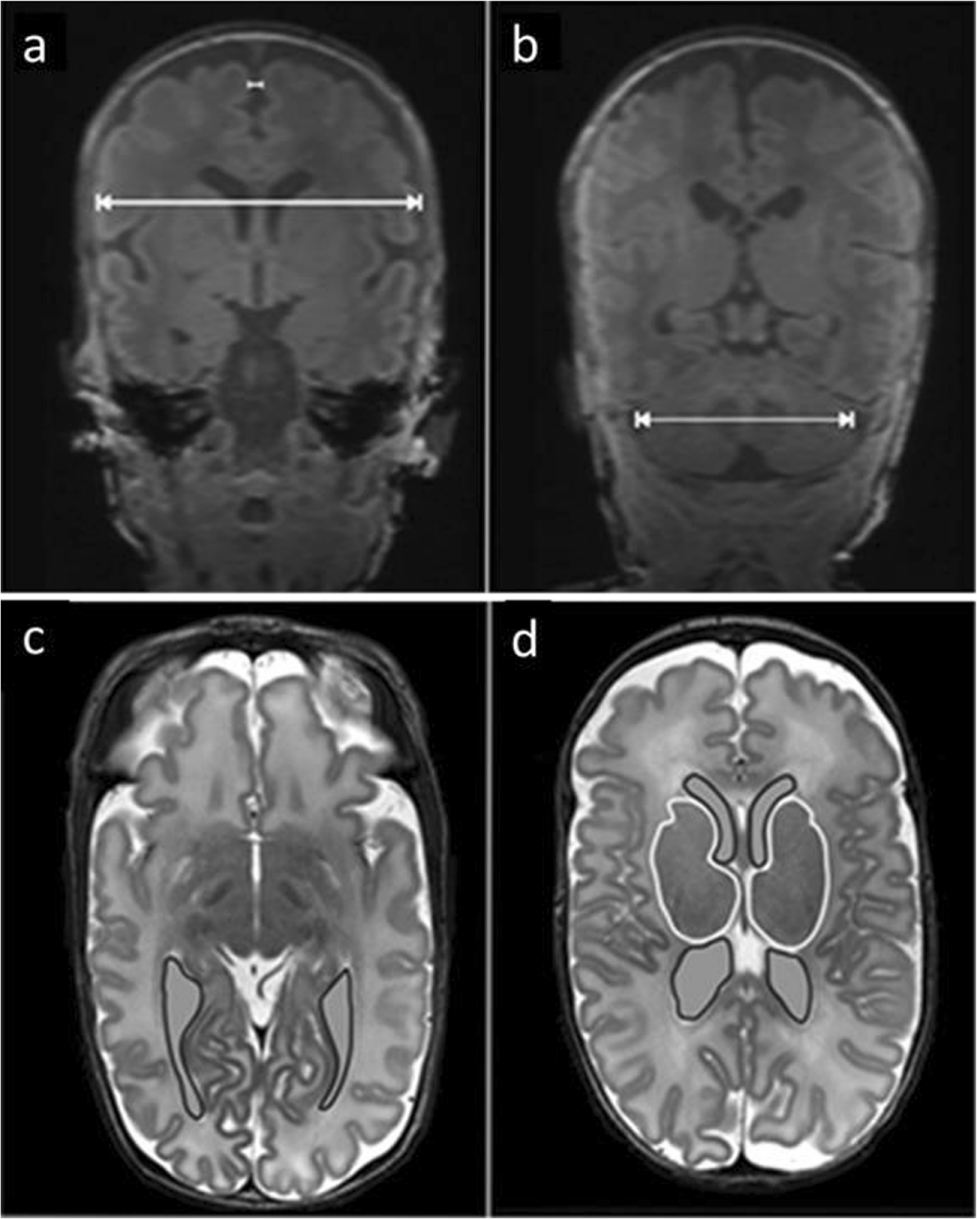
Examples of MRI measurements**. a, b** Measures of growth using brain metrics: interhemispheric distance (l—l; a), biparietal width (**←→**; **a**) and transcerebellar diameter (**←→**; **b**) on coronal T1-weighted images. These diameters were measured at the level of the 3rd ventricle, cochlea, and basilar artery (**a**) and at maximal width of the cerebellar diameter (**b**). **c**, **d** Volumetric measures of the lateral ventricles (black line; **c**, **d**) and the deep gray matter (white line; **d**) on an axial T2-weighted image, performed in every axial slice with lateral ventricles, respective deep gray matter visible

For MRI analysis, we combined and slightly modified the scores previously published by Kidokoro et al. [19, 20] (Supplementary Table 1). These scores for injury and altered brain development were summed up to a single total abnormality score (TAS) with a uniform grading system, 0–4 points/criterion (except gyration); gyration, 0–2 points; and maximum score, 22. In addition, our modification of the Kidokoro score focused on a more detailed description of extra-uterine brain development (myelination, gyration, ventricular dilation). Simple brain measurements (biparietal width (BPW), interhemispheric distance (IHD), transcerebellar diameter (TCD)), and volumetric analysis of the deep gray matter (DGM; basal ganglia and thalami), and lateral ventricles (LV) were used to support interpretation of white matter loss and development of DGM qualitatively, but were also considered separately for an independent analysis of predictive values (Fig. 2).

### Clinical factors

Clinical characteristics were collected from hospital records of each infant including gestational age, birth weight, head circumference, gender, multiple birth, courses of antenatal corticosteroids, chorioamnionitis, infection, IVH (intraventricular hemorrhage; including grade), patent ductus arteriosus (PDA; including treatment), bronchopulmonary dysplasia (BPD, divided into mild and severe), and necrotizing enterocolitis (NEC). Infection was defined by CRP (>1 mg/dl) and/or IL6 elevation (> 500 pg/ml), clinical deterioration, and additional pathologic laboratory parameters (e.g., thrombopenia, neutropenia). Ultrasound of the brain was routinely performed on day 1, 3, 5, 7, 14, 21, and 28 of life and every 4 weeks until discharge. IVH was graded using Papile’s classification system [26].

### Neurodevelopmental outcome

Neurodevelopmental outcome was assessed using Bayley Scales of Infant Development, 2nd Edition (BSID II), at a corrected age of 24 months including both mental and psychomotor developmental index [4].

### Statistical analysis

Data were analyzed with SAS Software, release 9.4 (SAS Institute Inc., Cary, NC, USA). Quantitative variables approximately normally distributed are presented as mean values (MW) together with standard deviations (SD) and ranges; for ordinally scaled or skewed data, median and range are given. For qualitative factors, absolute and relative frequencies have been assessed. In order to investigate the correlation between score and quantitative variable, Spearman correlation coefficient has been calculated. For quantitative outcomes, univariable and multiple regression analyses have been performed. The result of a statistical test has been considered as statistically significant for *p* < 0.05.

## Results

### Patients

We evaluated a total of 100 surviving preterm infants (< 32 weeks gestational age (GA); < 1500-g birth weight) born between January 2011 and December 2012. Sixty-two had to be excluded due to either inadequate quality of aEEG or MRI (for clinical details of the participants and excluded infants (drop-outs) see Supplementary Table 2). The study population consisted of 38 infants with a mean gestational age (GA) of 28.2 ± 2.3 weeks (Mean (M) ± Standard Deviation (SD); range 23.9–31.6) and a mean birth weight of 1093 ± 404 g (M ± SD; range 450–2085). Eighteen (47%) infants were female and 13 were multiples (34%). Seven children (18.4%) were born without any course of antenatal corticosteroids, the others received 1–3 courses of antenatal corticosteroids. Chorioamnionitis was histologically confirmed in nine cases (24%); 18 (47%) infants suffered from neonatal infections. Eight of the 38 patients (21%) and three of the cases with chorioamnionitis (33%) had early onset sepsis. The incidence of IVH (all grades) detected by ultrasound was 18% (*n* = 7) versus 29% (*n* = 11) detected by MRI (SWI-sequence). In none of the infants, CBH was diagnosed on ultrasound in contrast to three cases (8%) on MRI. A patent ductus arteriosus (PDA) was observed in 25 (69%) infants and four (11%) underwent surgical ligation. Twelve (32%) cases suffered from mild BPD (oxygen demand on day 28 of life) and two (5%) from severe BPD (oxygen demand at 36 weeks postmenstrual age). None of the infants showed necrotizing enterocolitis, but two (5.3%) developed focal intestinal perforation. As aEEG can be influenced by pH, blood pressure, analgesic or sedative medication, and mechanical ventilation, we analyzed patients’ charts for these parameters: Umbilical pH ranged between 7.13 and 7.54; two infants required low doses of catecholamines because of arterial hypotonia; and one infant benefited from epinephrine boli during reanimation. None of the infants without intubation received analgesic or sedative medication. In exceptional cases, medication was given for the procedure, accordingly aEEG recordings of the following 12 h were not evaluated. Twelve of the 38 patients were mechanically ventilated beyond surfactant application.

### aEEG analysis

aEEG tracings of 35 infants were evaluable for the BS on day 1 and 2 and of 30 infants on day 3 (Table 1). In 28 cases, aEEG records were available over the whole period of the first 72 h of life. The median values of the BS were 3.0 on day 1 (BS 1, range 1–8), 4.0 on day 2 (BS 2, range 1–8), and 5.5 on day 3 (BS 3, range 2–9), as well as 12.5 for the BS 72 h (range 5–25).

**Table 1:** Results of the aEEG recordings

	BS 1	BS 2	BS 3	BS 72 h
Median	3	4	5.5	12.5
Range	1–8	1–8	2–9	5–25
*n*	35	35	30	28

*n* number of patients; *BS 1,2,3* Burdjalov Score Day on day 1, 2, 3; *BS 72 h* Burdjalov Score of the first 72 h

### MRI analysis

Mean gestational age (GA) at scan was 40.0 ± 0.5 (M ± SD; range: 38.9–41.4) weeks. The median value of the total abnormality score (TAS) was 2 (range 0–10). Thirty-seven percent (*n* = 14) scored at least in one item of the injury score (periventricular leukomalacia (PVL), IVH and CBH), and 95% (*n* = 36) in at least one criterion of the altered developmental score (ventricular dilatation, gyration and myelination). Highest scores were obtained for ventricular dilatation (median: 1 point, range 0–3), followed by IVH (median: 0 point, range 0–3). Volumes of deep gray matter (basal ganglia and thalami) and lateral ventricles and measurements of biparietal width, interhemispheric diameter and transcerebellar diameter are shown in Table 2.

**Table 2:** Results of the MRI analysis

	GA MRI [wks]	DGMV [ml]	LV [ml]	BPW [mm]	IHD [mm]	TCD [mm]	TAS
Median	40	19.1	5.6	73.5	3.2	52	2
Range	38.9–41.4	10.9–22.1	2.7–16.9	63.3–82.9	1.6–7.8	38.3–57.8	0–10
*n*	38	38	38	38	38	38	38

*n* number of patients, *GA MRI* gestational age at MRI scan in weeks (wks), *DGMV* volume of the deep gray matter in ml, *LV* volume of the lateral ventricles in ml, *BPW* biparietal width in mm, *IHD* interhemispheric distance in mm, *TCD* transcerebellar diameter in mm, *TAS* total abnormality score

### Neurodevelopmental outcome

At 2 years’ corrected age (23 ± 3.7 completed months (M ± SD), range: 16–31 months), 27 children were assessed with the Bayley Scales of Infant Development II (BSID II, 71% follow-up rate). The remaining 11 infants were lost to follow-up due to frequent change of address or parental refusal to attend testing. One child did not complete Bayley testing and was only scored for mental developmental index (MDI). Scores of MDI or psychomotor developmental index (PDI) < 50 were considered as 40 to enable statistical analysis. The median value for MDI was 96 (range 62–124) and 95 (range 40–125) for PDI (Table 3).

**Table 3:** Results of the Bayley Scales II testing compared to calculated results following the formulas

	MDI	PDI	caMDI	caPDI
Median	96	95	92	95.5
Range	62–124	40–125	58–111	73–123
*n*	27	26	27	26

*n* number of patients, *MDI* mental developmental index, *PDI* psychomotor developmental index, *caMDI* calculated MDI following the formula, *caPDI* calculated PDI following the formula. PDI could not be assessed in 1 case. In addition, BS 1 was not recorded in 1 case, therefore calculation could only be done in 26 cases

### Relationship between brain function, maturation and brain structure

**Fig. 3:**
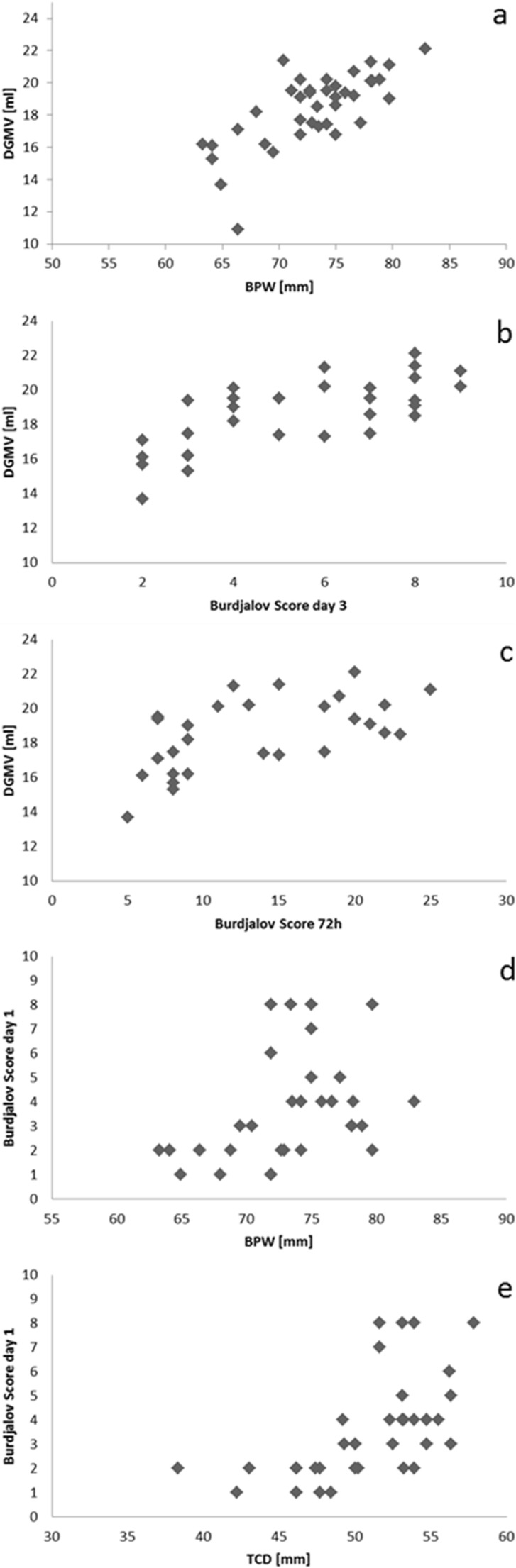
Significant relationship between brain function, maturation and brain structure. **a** Correlation between volume of deep gray matter (DGMV) and BPW (biparietal width); *p* < 0.0001, *r* = 0.7206. **b** Correlation between DGMV and Burdjalov Score day 3; *p* < 0.0001, *r* = 0.7216. **c** Correlation between DGMV and total Burdjalov Score 72 h; *p* = 0.0012, *r* = 0.5794. **d** Correlation between BPW and Burdjalov Score day 1; *p* = 0.0111, *r* = 0.4241. **e** Correlation between Burdjalov Score day 1 and transcerebellar diameter (TCD) and *p* = 0.0002, *r* = 0.5843. **a-e** Univariable analysis

Predictors for maturation of different brain structures are basal ganglia, thalamus, cerebellum, and global brain growth indicated by BPW. Diffuse white matter injury (dilatation of LV) and the TAS served as indicators for brain injury. Univariable analysis revealed the following predictors for reduced DGM volumes (Fig. 3): reduced BPW (*p* < 0.0001, *r* = 0.7206), low BS 3 (*p* < 0.0001, *r* = 0.7216), and low BS 72 h (summary of scores of day 1–3, *p* = 0.0012, *r* = 0.5794).

BPW represents brain growth. BS 1 (*p* = 0.0111, *r* = 0.4241) significantly correlated with BPW (univariable analysis; Fig. 3). The TCD was significantly associated with BS 1 (*p* = 0.0002, *r* = 0.5843, univariable analysis; Fig. 3).

### Predictors for neurodevelopmental outcome at corrected age of 24 months

**Fig. 4:**
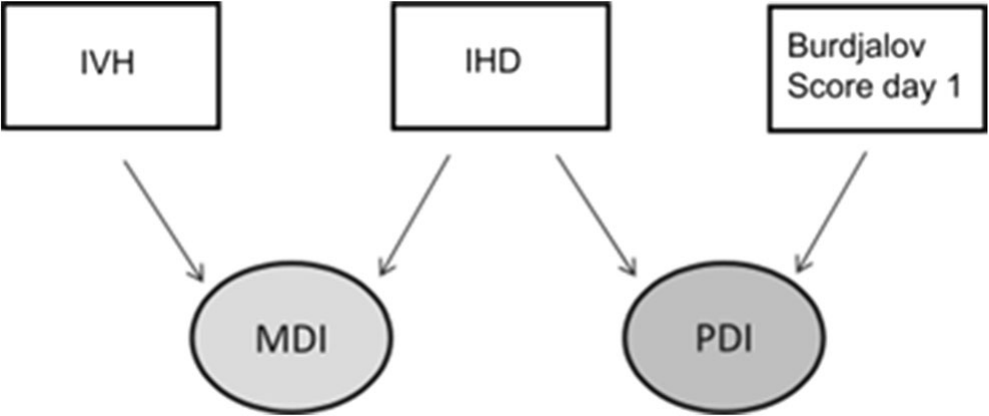
Independent predictors for MDI and PDI at corrected age of 24 months. IVH, intraventricular hemorrhage; MDI, mental developmental index; IHD, interhemispheric distance; PDI, psychomotor developmental index

Following multiple regression analysis, the MDI was significantly affected by the occurrence of IVH (*p* = 0.0060) and by reduced IHD (*p* = 0.0052). The goodness of this model may be quantified by *R*^2^ (*R*^2^ = 0.5716): 57% of the MDI variability is explained by the combination of IVH and IHD. PDI was associated with BS 1 (*p* = 0.0201) and IHD (*p* = 0.0142) (Fig. 4). About 36% of the PDI variability is explained by the combination of BS 1 and IHD in this model (*R*^2^ = 0.3578).

The multiple models used for statistical analysis of factors significantly associated (*p* < 0.05) with MDI or PDI at corrected age of 24 months resulted in the following mathematical equations:

- MDI = 73.3–29.7 · factor IVH + 6.1 · IHD [mm]

where factor IVH = 1 (IVH, any grade) and factor IVH = 0 (no IVH). Occurrence of IVH leads to subtraction of 29.7 points.

- PDI = 54.7 + 5.7 · IHD [mm] + 5.24 · BS 1

Table 3 represents the predictive value of the mathematical equation in anticipating MDI and PDI scores. It is obvious that both scores, but especially PDI, are also dependent from other factors as the prediction is not true in every case.

## Discussion

Injurious insults, therapeutic interventions, and stress may alter spontaneous neuronal activity known to be crucial for survival of brain cells, axonal outgrowth, and formation of neuronal circuits [13, 17, 33]. This explains the still existing risk of long-term neurologic impairment following preterm birth. However, biomarkers to identify children at high risk are rare. The present study describes an association between early brain function monitored by amplitude-integrated electroencephalography (aEEG) in the first 72 h of life and altered brain growth on magnet resonance imaging (MRI) at term equivalent age (TEA) and the consequences for neurodevelopmental outcome at 24 months.

Benders et al. found a positive correlation of increased brain activity during the first days of life with brain growth until term age [5], stressing the importance of specifically deep gray matter (DGM) growth on the development of cortical networks [22]. In their analysis, they used volumetric measures and found the total cerebral brain volume (including cerebrospinal fluid) most useful to describe global brain growth [5]. The aberrant development of the DGM is recognized as a remote effect to less neuronal activity resulting in reduced connectivity with other structures [6]. Our results obtained with easy scoring systems for analysis of aEEG and conventional MRI at TEA revealed a reduced biparietal width (BPW), which was influenced by early brain activity. By assessing brain injury Song et al. showed that abnormal aEEG patterns can predict white matter damage and long-term handicap in preterm infants. However, gray matter injury and brain growth were not addressed [32]. To relate aEEG activity to both brain development and injury, we modified the Kidokoro scoring system to a total abnormality score (TAS) by adding more detailed items for maturation and tested its ability to predict outcome.

A high prevalence of either injury (37%) or altered maturation (95%) was detected, severe patterns of injury (IVH > II°, cystic PVL) were less common (11%, 0%), and TAS was rather low with a median of 2 (range 0–10). The levels and distributions of scores are in accordance with Kidokoro et al. [19, 20] where at least 33% of infants showed injury and 97% at least one item of the developmental score when applying the modified score (myelination delay, dilated lateral ventricles ,and gyral maturation). TAS had no predictive value, most likely explained by the small sample and an underestimation of subtle alterations by TAS.

In the present study, IVH was an independent predictor of the mental developmental index (MDI, BSID II). Higher rates of intraventricular hemorrhage on MRI than the USA may be explained by the evaluation of hemorrhage with the SWI sequence. SWI is more sensitive in detecting hemoglobin, iron, and small bleedings than ultrasound or conventional MRI sequences [12, 16]. The influence of IVH on neurodevelopmental outcome, predominantly low grade IVH, is controversially discussed. Patra et al. reported lower MDI scores at 20 months’ corrected age in extremely low birth weight infants with grade I–II IVH [28] while Ann Wy et al. did not see low-grade IVH as an independent risk factor for poor outcome at 36 months, 8, and 18 years [3]. However, the majority of studies link higher grade IVH with major neurodevelopmental disabilities [2]. Although ventricular dilatation is common and accounts for the majority of scoring points in the qualitative analysis, it is not predictive for outcome. Ventricular dilatation alone does not seem to increase the risk for adverse outcome, but in conjunction with other brain abnormalities, the risk for motor and cognitive impairment is increased [24].

Interhemispheric diameter (IHD) was an independent predictor of psychomotor developmental index (PDI, BSID II) and MDI in our study population potentially indicating altered brain growth while BPW was not predictive. Increased IHD indicates impaired brain growth compared to head/skull as indicated by BPW [20]. However, in this study, multiple regression analysis revealed that small head circumference on MRI was associated with short IHD (*p* = 0.0139), indicating a proportional growth alteration. In contrast, Kidokoro et al. described an independent association between decreased BPW and increased IHD with perinatal risk factors and cognitive outcome in two of three cohorts (New Zealand, Australia, USA) and described IHD as an indicator for disproportional brain growth and reduced BPW for generally impaired growth [20]. Differences in measurement techniques (choice of planes and landmarks) and inhomogeneity of cohorts may account for these contradicting results. The range of postmenstrual age at MRI was smaller in our cohort (38.9–41.4 weeks) compared to 37–42 weeks in the Kidokoro study, thus correction for age at scan was not performed in our study. The smaller number of cases in the present study and the higher median gestational age at scan may also account for different findings. In a cohort of 239 extremely preterm infants, Brouwers et al. could not underline the prognostic value of the Kidokoro score [9]. Altogether, MRI scoring systems and their validity for neurodevelopmental outcome should be analogously tested in different populations and various departments of neonatology.

In the present study, Burdjalov Score on day 1 was associated with transcerebellar diameter and with biparietal width. On day 3 and in the first 72 h, Burdjalov Score correlated with deep gray matter volumes. This striking association between early brain function and brain maturation at term may be attributed to the co-occurrence of injurious stimuli and ongoing developmental processes of proliferating brain cells (subplate neurons, premyelinating oligodendrocyts, and migrating neurons) resulting in either cell loss or failure to mature [8]. Long-term outcome can be predicted by EEG already in the first postnatal hours in very preterm infants [7, 36, 37]. In the present study, the Burdjalov score of the first day of life is an independent predictor of the PDI. Further, we identified the combination of aEEG and MRI at TEA as a potentially predictive biomarker for neurologic impairment after preterm birth. Nevertheless, their predictive value has to be evaluated in other cohorts and higher numbers of patients.

We conclude that early postnatal aEEG combined with cerebral MRI at TEA contribute to the prediction of neurodevelopmental outcome in preterm infants at 2 years of corrected age. Our study supports a clear relationship between early activity and brain growth processes. Therefore, aEEG monitoring in the first 72 h of life and MRI at TEA can be used to identify preterm infants at high risk for neurodevelopmental impairment.

## Electronic supplementary material

ESM 1 (DOCX 19.4 kb)

ESM 2 (DOCX 15.7 kb)

## Abbreviations

aEEG: Amplitude-integrated electroencephalography
BPD: Bronchopulmonary dysplasia
BPW: Biparietal width
BS: Burdjalov Score
BS 1,2,3: Burdjalov Score on day 1, day 2, or day 3
BS 72 h: Burdjalov Score day 1–3 (first 72 h of life)
BSID II: Bayley Scales of Infant Development, 2nd Edition
CBH: Cerebellar hemorrhage (s)
DGM (V): Deep gray matter (volume)
h: Hours
IHD: Interhemispheric distance
IVH: Intraventricular hemorrhage
LV: Lateral ventricles
M: Mean
MDI: Mental developmental index
MRI: Magnetic resonance imaging
n: Number of patients
NEC: Necrotizing enterocolitis
NICU: Neonatal intensive care unit (s)
PDA: Patent ductus arteriosus
PDI: Psychomotor developmental index
PVL: Periventricular leukomalacia
SD: Standard deviation
TAS: Total abnormality score
TCD: Transcerebellar diameter
TEA: Term equivalent age
