# Supplementary material for: Relationship between brain function (aEEG) and brain structure (MRI) and their predictive value for neurodevelopmental outcome of preterm infants
Source: Eur J Pediatr. 2018 May 22;177(8):1181–9. doi: 10.1007/s00431-018-3166-2 (PMC6061051; doi:10.1007/s00431-018-3166-2)
Supplement: Supplementary file 1 — (DOCX 19.4 kb) [file 431_2018_3166_MOESM1_ESM.docx]

**Table Supplement**

**Supplementary Table 1: Comparison of Kidokoro Scores, its modification and list of subjects affected**

| **Grading system according to Kidokoro 2014**  ***Injury classification*** | **Modified Total Abnormality Score**  ***Injury score*** | **subject of modification** |
| --- | --- | --- |
| **PVL** (grade 1-4), (T1, T2)  **1 =** punctates ≤ 3 mm unilat. /bilat.  and < 3 lesions per hemisphere  **2 =** punctate lesions ≤ 3 mm in  corticospinal tracts or ≥ 3 lesions  per hemisphere  **3 =** extensive lesions along the wall of  LV, high signal T1  **4 =** cystic lesions in periventricular wm | **PVL** (grade 0-4), (T1, T2)  **0 =** nil (no signs for lesions in  periventricular wm) on T1 and T2  **1 =** unilat./bilat. punctate lesions ≤ 3 mm  and < 3 lesions per hemisphere  **2 =** lesions in bilat. cortico-spinal tracts or  punctate lesions > 3mm and/or ≥ 3  lesions per hemisphere  **3 =** extensive lesions along the wall of LV,  high sign on T1  **4 =** cystic lesions in periventricular wm  on T1 and T2 | Grade 0  (to indicate no injury)  Grade 2  lesions > 3 mm; |
| **IVH** (grade 1-4), (T1, T2)  **1 =** hemosiderin deposits,  posthemorrhagic cysts within  caudo-thalamic notch  **2 =** hemosiderin deposits outside  notch, along ventricle wall without  VD  **3 =** VD > 97th  percentile with evidence of  previous ventricular haemorrhage  **4 =** parenchymal hemorrhagic lesions  or posthemorrhagic cystic  encephalomalacia | **IVH** (grade 0-4), (SWI,T2)  **0 =** nil (no hemosiderin deposits)  **1 =** hemosiderin deposits, posthemorrhagic  cysts within caudo-thalamic notch  **2 =** hemosiderin deposits outside notch,  along ventricle wall without VD  **3 =** obvious VD with  evidence of previous ventricular  haemorrhage  **4 =** parenchymal hemorrhagic lesions or  posthemorrhagic cystic encephalopathy | Grade 0  (to indicate no injury)  VD visualized in various planes |
| **CBH** (grade1-4), (T1,T2)  **1 =** unilat. punctate lesions ≤ 3 mm  **2 =** bilat. punctate lesions  **3 =** unilat. lesions > 3mm  **4 =** extensive lesions bilat. | **CBH** (grade0-4),(SWI, T2)  **0 =** nil (no haemorrhage)  **1 =** unilat. haemorrhage ≤ 3 mm, ≤ 3 lesions per  hemisphere  **2 =** bilat. haemorrhage ≤ 3 mm, ≤ 3 lesions per  hemisphere  **3 =** unilat. punctate lesions >3 mm and / or ≥ 3  lesions per hemisphere  **4 =** extensive lesions bilat., ≥ 1 lesion > 3mm | Grade 0  (to indicate no injury)  number of lesions account for grading |
| ***Brain growth/development***  ***Kidokoro 2013/Nguyen The Tich 2009*** | ***Development Score*** | **subject of modification** |
| **Biparietal width (BPW)**  (maximal horizontal brain width of frontal lobes)  Coronal images (T2) with bilat. cochlea and basilar truncus as standard landmarks  **Interhemispheric distance (IHD)**  (horizontal distance between the tops of the crowns of the superior frontal gyri) | **Biparietal width (BPW)**  (maximal horizontal brain width of frontal lobes)  Coronal images (T1) with bilat. cochlea and/or basilar truncus as standard landmarks  **Interhemispheric distance (IHD),** (T1)  (horizontal distance between the top of the crowns of the superior frontal gyri) | Measurements done on T1 weighted images  no contribution to TAS |
| ***Additional grading of subtle abnormalities***  ***Kidokoro 2013/Inder 2003*** |  |  |
| **Myelination** (grade 0-2), (T1)  **0 =** PLIC & corona radiata  **1 =** only PLIC  **2 =** minimal – no PLIC | **Myelination**, (grade 0-4), (T1)  **0 =** ≥ 1/3 PLIC + centrum semiovale  **1 =** ≥ 1/3 PLIC + optic radiation, not  centrum semiovale  **2 =** ≥ 1/3 PLIC, but not in centrum  semiovale and optic radiation  **3 =** ≤ 1/3 PLIC, clearly visible  **4 =** no myelin in PLIC | Additional subjects to describe potential diffuse / subtle WM abnormalities |
| **Dilated LV**, (grade 0-3), (T1)  **0 =** both sides ventricular diameter  (VDi) < 7,5 mm)  **1 =** one side 7,5 mm < VDi < 10 mm  **2 =** both sides 7,5 mm < VDi< 10 mm  or one side > 10 mm  **3 =** both sides > 10 mm | **Ventricular dilatation (VD)**, (grade 0-4), (T2)  **0 =** nil, slim ventricles both sides  **1 =** mild dilatation, with modelling to gyri  **2 =** moderate VD  **3 =** severe VD  **4 =** porencephalie | Qualitative description completed by 3D-volumetry |
| **GM/cerebellum score** |  |  |
| **Gyral maturation**, (grade 0-2), (T2)  **0 =** delay < 2 wks  **1 =** 2<delay<4 wks  **2 =** delay > 4 wks | **Gyration**, (grade 0-2), (T1)  **0 =** nil ( 40 wks, tert. gyri sulci inf. temp and  inf. occip.)  **1 =** (36-38 wks, additional sec. gyri transv. and  inf.- temp.)  **2 =** (34-36 wks, present marg. sulcus,  paracentral gyrus, sec. gyri front) | none |
| **Volume reduction – deep gray matter area (DGMA)**, (grade 0-3), (T2)  **0 =** corr. DGMA (cDGMA) > 9,5  **1 =** 9,5 > cDGMA >8,5  **2 =** 8,5> cDGMA >7,5  **3 =** 7,5 > cDGMA | **Deep grey matter** no grading, (T2)  3D-volumetry | 3D-volumetry,  no contribution to TAS |
| **Volume reduction – cerebellum**, (grade 0-3), (T2)  (maximal horizontal distance, level of the atria (plexus choroid apparent)  **0 =** corr. Transcerebellar diameter  (cTCD) > 50 mm  **1 =** 50 mm > cTCD > 47 mm  **2 =** 47 mm > cTCD < 44mm  **3 =** cTCD< 44mm | **Transcerebellar diameter (TCD)**,  (no grading), (T1)  (maximal horizontal distance, level of the atria (plexus choroid apparent) | none,  no contribution to TAS |

PVL = periventricular leukomalacia, IVH = intraventricular haemorrhage, WM = white matter, GM = grey matter, LV = lateral ventricles, VD = ventricular dilatation, CBH = cerebellar haemorrhage, TAS = total abnormality score; PLIC = posterior limb of internal capsule, VD = ventricular dilatation, wks = weeks, TCD = transcerebellar diameter, DGMA = deep grey matter area, c = corrected
