# Supplementary material for: Relationship between brain function (aEEG) and brain structure (MRI) and their predictive value for neurodevelopmental outcome of preterm infants
Source: Eur J Pediatr. 2018 May 22;177(8):1181–9. doi: 10.1007/s00431-018-3166-2 (PMC6061051; doi:10.1007/s00431-018-3166-2)
Supplement: Supplementary file 2 — (DOCX 15.7 kb) [file 431_2018_3166_MOESM2_ESM.docx]

**Table Supplement**

**Supplementary Table 2: Clinical details of the participants and of infants not eligible for the study (drop-outs)**

| **Parameters** | **Participants (n=38)** | **Drop-outs (n=63)** | **P value** | **Statistical test** |
| --- | --- | --- | --- | --- |
| gestational age at birth [weeks]  mean ± SD (range) | 28.2 ± 2.3  (23.9 - 31.6) | 28.8 ± 2.2  (23.3 - 31.7) | 0.3097 | *U*-test |
| birth weight [g]  mean ± SD (range) | 1093 ± 404  (450 – 2085) | 1169 ± 353  (480 – 1860) | 0.3218 | t-test |
| IVH** - Yes/No [n]  Grade I [n] (%)  Grade II [n] (%)  Grade III [n] (%)  parenchymal hemorrhage [n] (%) | 7/31  4  3  0  0 | 3/59  1  0  2  2 | Yes/no:  0.0397  Grades:  0.0377 | Fisher´s Exact test  *U*-test |
| PVL [n] (%)  No  Yes | 38 (100)  0 (0) | 61 (98.4)  1 (1.6) | 1.000 | Fisher´s Exact test |
| BPD [n] (%)  No  Yes | 36 ()  2 (5) | 56 (90.3)  6 (9.7) | 0.7066 | Fisher´s Exact test |
| NEC [n] (%)  No  Yes | 38 (100)  0 (0) | 60 (96.8)  2 (3.2) | 0.5240 | Fisher´s Exact test |
| FIP [n] (%)  No  Yes | 36 (94.7)  2 (5.3) | 61 (98.4)  1 (1.6) | 0.5556 | Fisher´s Exact test |

26 ()00)
5). trated in the supplementary Table 2nd the participation on Bayley test with 24 months

IVH = intraventricular hemorrhage, based on cranial ultrasound, grading according to Papile, BPD = bronchopulmonary dysplasia, defined as oxygen demand at 36 weeks postmenstrual age, NEC = necrotizing enterocolitis, FIP = focal intestinal perforation, *U*-test = Mann-Whitney-*U*-test
